# A dataset of pomegranate growth stages for machine learning-based monitoring and analysis

**DOI:** 10.1016/j.dib.2023.109468

**Published:** 2023-08-03

**Authors:** Jifei Zhao, Rolla Almodfer, Xiaoying Wu, Xinfa Wang

**Affiliations:** School of Computer Science and Technology, Henan Institute of Science and Technology, Xinxiang, Henan Province, 453003, China

**Keywords:** Pomegranate growth period detection, Image classification, Image Detection, Feature extraction

## Abstract

Machine learning and deep learning have grown very rapidly in recent years and are widely used in agriculture. Neat and clean datasets are a major requirement for building accurate and robust machine learning models and minimizing misclassification in real-time environments. To achieve this goal, we created a dataset of images of pomegranate growth stages. These images of pomegranate growth stages were taken from May to September from an orchard inside the Henan Institute of Science and Technology in China. The dataset contains 5857 images of pomegranates at different growth stages, which are labeled and classified into five periods: bud, flower, early-fruit, mid-growth and ripe. The dataset consists of four folders, which respectively store the images, two formats of annotation files, and the record files for the division of training, validation, and test sets. The authors have confirmed the usability of this dataset through previous research. The dataset may help researchers develop computer applications using machine learning and computer vision algorithms.

**Specifications Table**

**Table:** 

Subject	Agriculture Engineering, Computer Vision and Pattern Recognition, Data Science, Artificial Intelligence
Specific subject area	Machine learning-based image detection and classification of pomegranate growth stages.
Data format	Raw: HEIC Conversion: jpg Annotation: XML and TXT
Type of data	Image
Data collection	Collect images of pomegranates throughout their growth cycle. The iPhone's high-resolution rear camera is used to capture images of pomegranates at different stages of growth. The original images were in HEIC format with a size of 3024*4032 pixels. These pomegranate growth stage images were taken in a sunny environment in a pomegranate orchard at Henan University of Science and Technology in China, with coordinates at 35.3602°N and 113.9505°E. The images were taken from May to September, approximately every 10-15 days. The images were further categorized into five periods: Bud, Flower, Early-fruit, Mid-growth, and Mature, resulting in a total of 5857 images. Subsequently, the images were converted to JPG format with a size of 640*480 pixels. Manual annotation was performed to label the images with category and location information, and the annotations were saved in XML and TXT formats for use with different models. The dataset was split into training, validation, and testing sets suitable for machine learning. The folder size for the dataset is 800 MB, and a RAR file was provided for convenient downloading.
Data source location	Institution: Henan Institute of Science and Technology City: Xinxiang, Henan Country: China Latitude and longitude (and GPS coordinates, if possible) for collected samples/data: 35.3602°N and 113.9505°E, Altitude: 75 msl
Data accessibility	Repository name: Mendeley Data Data identification number: 10.17632/kgwsthf2w6.4 Direct URL to data: https://data.mendeley.com/datasets/kgwsthf2w6

## 1 Value of the Data

• The dataset helps researchers to develop computer applications using machine learning and computer vision algorithms on the one hand. On the other hand, studying the different growth stages of pomegranate can effectively monitor the condition of each growth stage pomegranate and help farmers to detect abnormal growth conditions early to help reduce economic losses.
• The dataset retains images of all stages of pomegranate growth. For one, it can be used by computer-related researchers to test and compare different computer vision and image processing classifiers to develop algorithms, programs, and other applications. Secondly, the dataset helps to build high-quality pomegranate management applications which are beneficial for farmers, agriculture, wholesalers, and pomegranate export companies. Third, a portion of the pomegranate plant image dataset was taken from a wider field of view, so plant physiologists may need to perform an in-depth analysis.
• The dataset is released for public use and readily available for download, so researchers can place the dataset directly into a machine learning system without any further validation/pre-processing. Computer-related researchers can perform the identification, and detection of pomegranates and the staging of their growth period. Texture descriptions and color descriptions of different types of pomegranates are used to support numerous feature selectors and feature extractors.
• We develop the first dataset of images of the growth stages of pomegranates in China, the largest pomegranate-growing country in the world. All 5857 images were taken manually from the pomegranate orchard by a cell phone camera and then tagged by human experts.
• Detecting the number of pomegranate flowers in the data set will help fruit farmers to calculate the fruiting rate of pomegranates and help them to estimate the yield of pomegranates. Testing the ripeness of pomegranates will help fruit farmers to predict when they will ripen.
• Although this dataset only contains images of Chinese pomegranates, given its large size, this could easily be used in a migration learning setup to detect other species of pomegranates.

## 2 Data Description

The dataset described in this article contains 5857 pomegranate images at different growth stages, which are classified into five periods: bud, flower, early-fruit, mid-growth and ripe. Researchers can use different computer vision and image processing algorithms to recognize and detect these two versions of the dataset. All images were manually taken with an iPhone XR camera on the campus of Henan University of Science and Technology with the help of pomegranate research experts. All original images have a resolution of 72 dpi, with a width of 4032 pixels and a height of 3024 pixels. The adjusted image size is 640*480. The data were collected as shown in Table 1.

**Table 1:** Brief description about the data collection.

No.	Particulars	Description
1	Fruit	Pomegranate
2	Number of growing periods considered	5
3	Photo Time	May 1 - September 1, 2022, shooting every 10-15 days.
		The times are all sunny daytime hours.
4	Geographical location	35.3602°N 113.9505°E, Altitude: 75 msl
5	Climatic	Sunny day
6	Temperature	28-38°C

Our original dataset contains a total of 5857 data images in HEIC format, which can be used for detecting the growth cycle of pomegranates. Next, we used iMazing Converter, a software developed by DigiDNA Sàrl, to convert all pomegranate images into the jpg format.

And for these 5857 images, we used the open-source annotation software LabelImg to annotate the pomegranate dataset, dividing the pomegranates into five different categories: bud, flower, early-fruit, mid-growth, and mature, as shown in Table 2.

**Table 2:** Description of the dataset for the growth period of pomegranate.

Class	Bud	Flower	Early-fruit	Mid-growth	Mature	All
Num.	1245	1243	1007	1259	1103	5857

### 2.1 Description of Pomegranate Growth Stages

#### 2.1.1 Bud

**Fig. 1:**
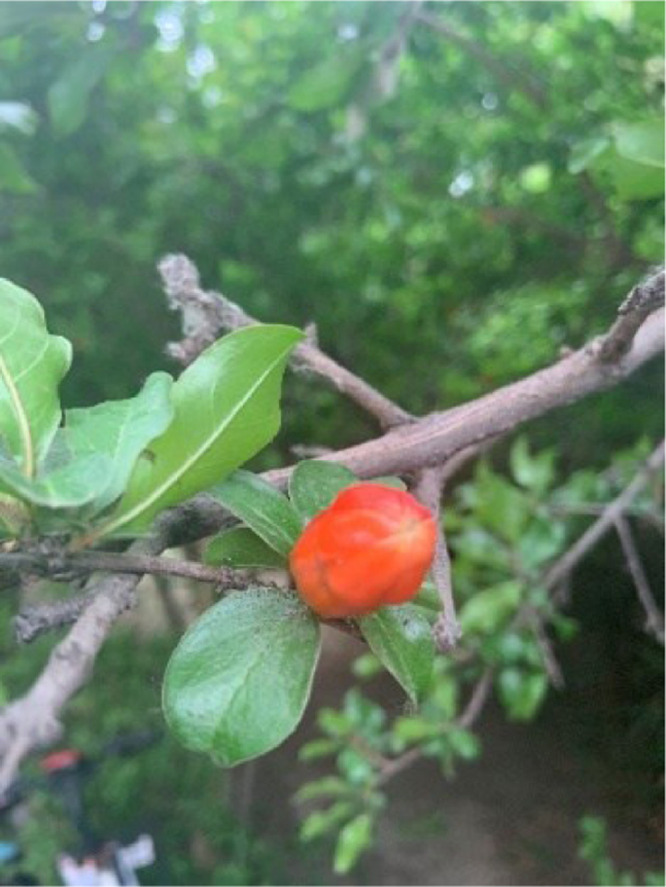
The pomegranate bud growth stage.

When the size of a single bud is about the size of a mung bean, it can be determined that the pomegranate has entered the bud stage, as shown in Fig. 1. The period from the bud stage to the flowering stage is 5 to 12 days. In spring, the bud stage of pomegranates can last for 20 to 30 days due to the low temperature.

#### 2.1.2 Flower

**Fig. 2:**
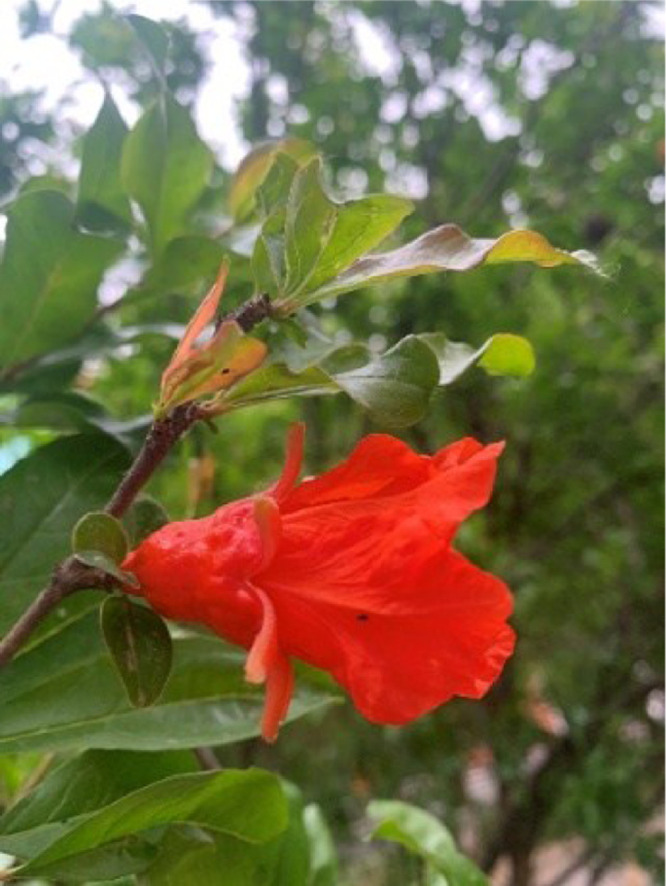
The pomegranate flower growth stage.

Pomegranate flowers can be divided into bisexual types. They grow 1 to several at the top of small branches or in leaf axils with very short peduncles. The calyx is bell-shaped, thick, and red, about 2-3 cm long. As the flower bud grows, the calyx starts to separate, with 5-7 fissures at the top, the lobes spreading outward, and the shape of the lobes being ovate-triangular, about 8-13 mm long. After 3-5 days of separation, the corolla opens, with the same number of petals as calyx lobes, opposite each other and born inside the calyx tube. The petals are inverted egg-shaped, about 13-15 cm long and 1-2 cm wide, with a rounded tip as shown in Fig. 2. They can also be divided into trumpet-shaped and tubular-shaped.

#### 2.1.3 Early-fruit

**Fig. 3:**
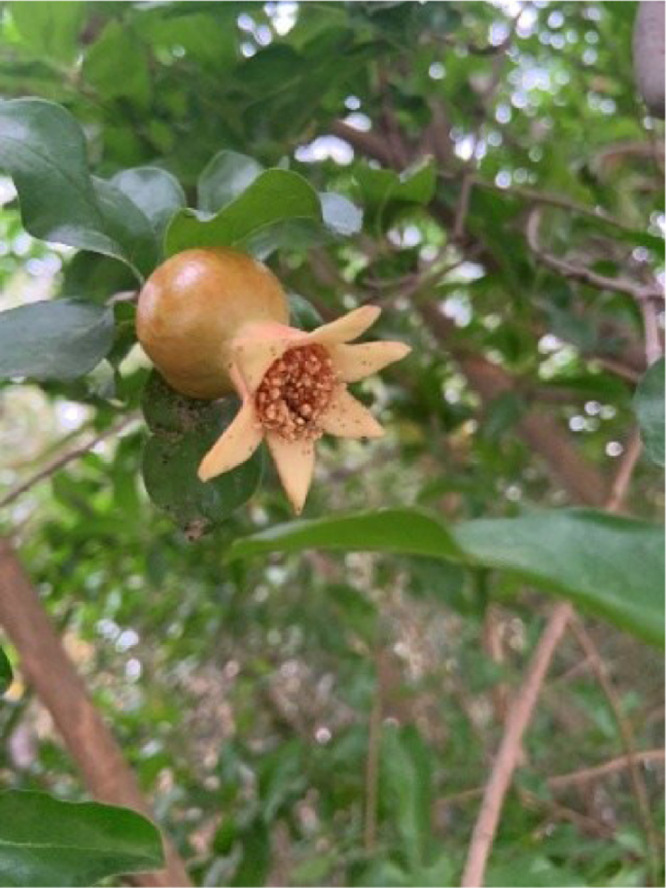
The pomegranate early-fruit growth stage.

During the fruit setting period, the petals fall off and the fruit starts to enlarge, with its color changing to yellow-brown, as shown in Fig. 3.

#### 2.1.4 Mid-growth

**Fig. 4:**
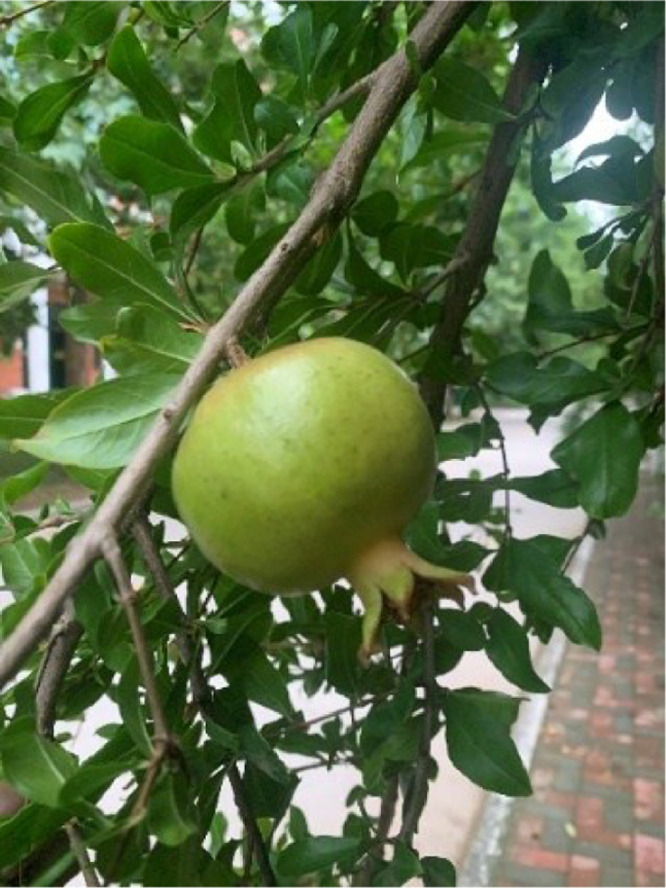
The pomegranate mid-growth growth stage.

The color change period (pre-harvest enlargement period) mostly occurs 4-5 weeks before harvest, during which the volume increases rapidly, accounting for 22.6% to 27.5% of the total growth. The color of the fruit changes to green, as shown in Fig. 4.

#### 2.1.5 Mature

**Fig. 5:**
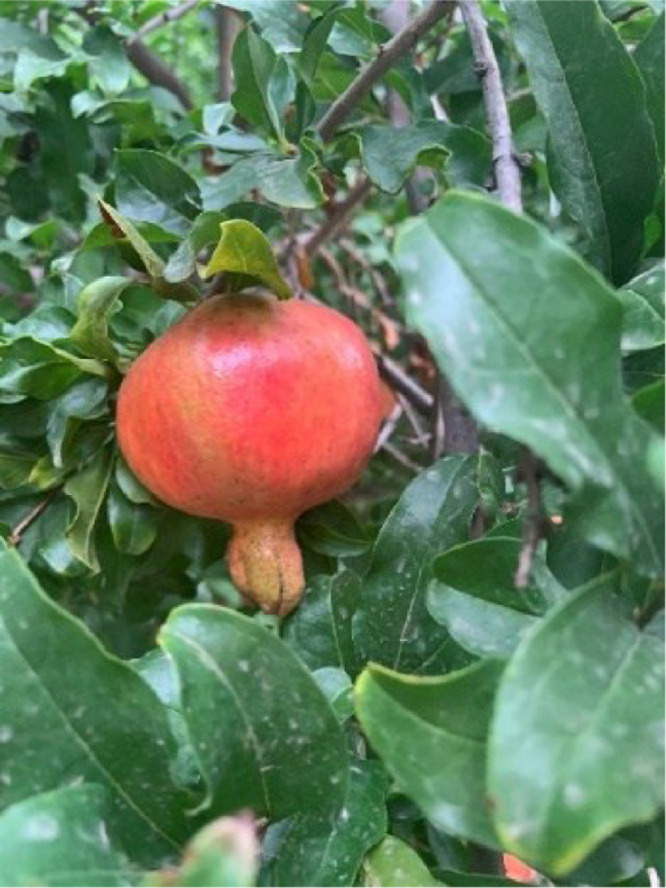
The pomegranate mature growth stage.

After ripening, the fruit skin changes from green to yellow, and colored varieties are fully colored (the pomegranates in this dataset are bright red, as shown in Fig. 5). The fruit surface becomes shiny, and the fruit becomes a large, multilocular, and polyspermic berry, with numerous seeds in each locule. The fruit ridges begin to appear, and the seeds are plump, juicy, sweet, and sour, which is the edible part. The inner seed coat is keratinous, and can also become soft and degenerate, known as soft-seed pomegranate. The soluble solids content of the fruit juice reaches the inherent concentration of the variety.

### 2.2 Significance of the Dataset

The dataset we propose consists of 5 categories, each representing a different growth stage that requires specific cultivation techniques to ensure healthy and rapid growth of pomegranates. The considerations and cultivation techniques for each growth stage are listed in Table 3.

**Table 3:** Description and cultivation techniques during the growing period.

Class	Description	Cultivation techniques
Bud	After pomegranate trees sprout, many buds often sprout from the main trunk, main branches, and various secondary branches. However, new shoots that sprout from the base of the main trunk, the bifurcation of the branches, and the back of the branches tend to grow more vigorously. These new shoots generally do not have flower buds and are considered ineffective branches that pose a potential threat of ``flower and fruit snatch''	Researchers can use this dataset to detect the current bud stage of pomegranates and inform farmers to remove all non-flower buds on pomegranate trees in the fruiting stage, in order to concentrate nutrients on ensuring normal flowering and fruit setting of the head crop. After the head crop has set fruit and the quantity of fruit meets the production requirements, the later buds can be retained to facilitate the expansion of the canopy and filling of the space between branches.
Flower	The trumpet-shaped flowers of pomegranate trees are not healthy in development and cannot successfully bear fruit. Instead, they will consume a lot of nutrients, so we must remove them as early as possible. During the pomegranate's current budding period, we can clearly distinguish between tubular and trumpet-shaped flowers, and it is best to thin the flowers at this stage. Thinning the flowers once every 10-15 days can increase production by more than 20% in the same year.	Researchers can use this dataset to detect and classify trumpet-shaped and tube-shaped flowers. By comparing it with the pomegranate tree in the current budding stage, they can identify whether a budding pomegranate will grow into a trumpet-shaped flower. If it will, then the pomegranate should be removed during the budding stage and the tube-shaped flowers should be preserved.
Early-fruit	The fruit-setting rate of pomegranates is not necessarily high as only fully developed flowers can set fruit. It is important to focus on pest and disease control during the early-fruit stage, which includes the periods before and after fruit setting.	This dataset can be used to measure the fruit-setting rate of pomegranates in orchards and also provides guidance for pest and disease control during the pomegranate growth process. By monitoring the growth of pomegranates, the occurrence of pest and disease can be effectively prevented.
Mid-growth	The mid-growth stage is a crucial phase in the pomegranate growth process. If the fruits on the pomegranate tree are too densely packed, it can affect the size and quality of the fruits. Additionally, the mid-growth stage is a vulnerable period for the pomegranate tree to be attacked by pests and diseases.	It is necessary to thin the fruits at the appropriate time by removing excessive fruits and retaining an adequate number of fruits. This promotes the proper development and high-quality yield of the remaining fruits. By implementing proper fertilization, irrigation, pruning, and pest and disease control measures, the healthy development and high productivity of pomegranate fruits can be facilitated.
Mature	The mature fruits should be harvested before the rainy season, and harvesting is prohibited during cloudy and rainy weather.	The mature fruits should be harvested before the rainy season, and harvesting is prohibited during cloudy and rainy weather.

Our dataset comprises five growth stages of pomegranates: Bud, Flower, Early-fruit, Mid-growth, and Mature stages. Unlike other agricultural datasets, our dataset captures images throughout the pomegranate growth process, filling the gap of limited publicly available data on pomegranate growth stages. The primary contribution of this dataset lies in providing an opportunity to study the different growth stages of pomegranates, facilitating the monitoring of their growth status and assisting farmers in early detection of abnormal growth conditions to minimize economic losses.

In summary, our dataset has been manually annotated to create a usable pomegranate growth stage classification dataset, offering a resource for computer scientists engaged in scientific research and model development. Furthermore, it provides assistance to agricultural workers, who can utilize real-life images of pomegranate growth stages for research purposes and analyze the actual growth status of the fruit based on specific images. Ultimately, through the research and development efforts of computer scientists, this dataset can offer practical cultivation recommendations for pomegranate farmers at different growth stages, helping them enhance their management practices in pomegranate cultivation. The contribution of this dataset holds significant real-world significance.

### 2.3 Description of Dataset Folders

**Fig. 6:**
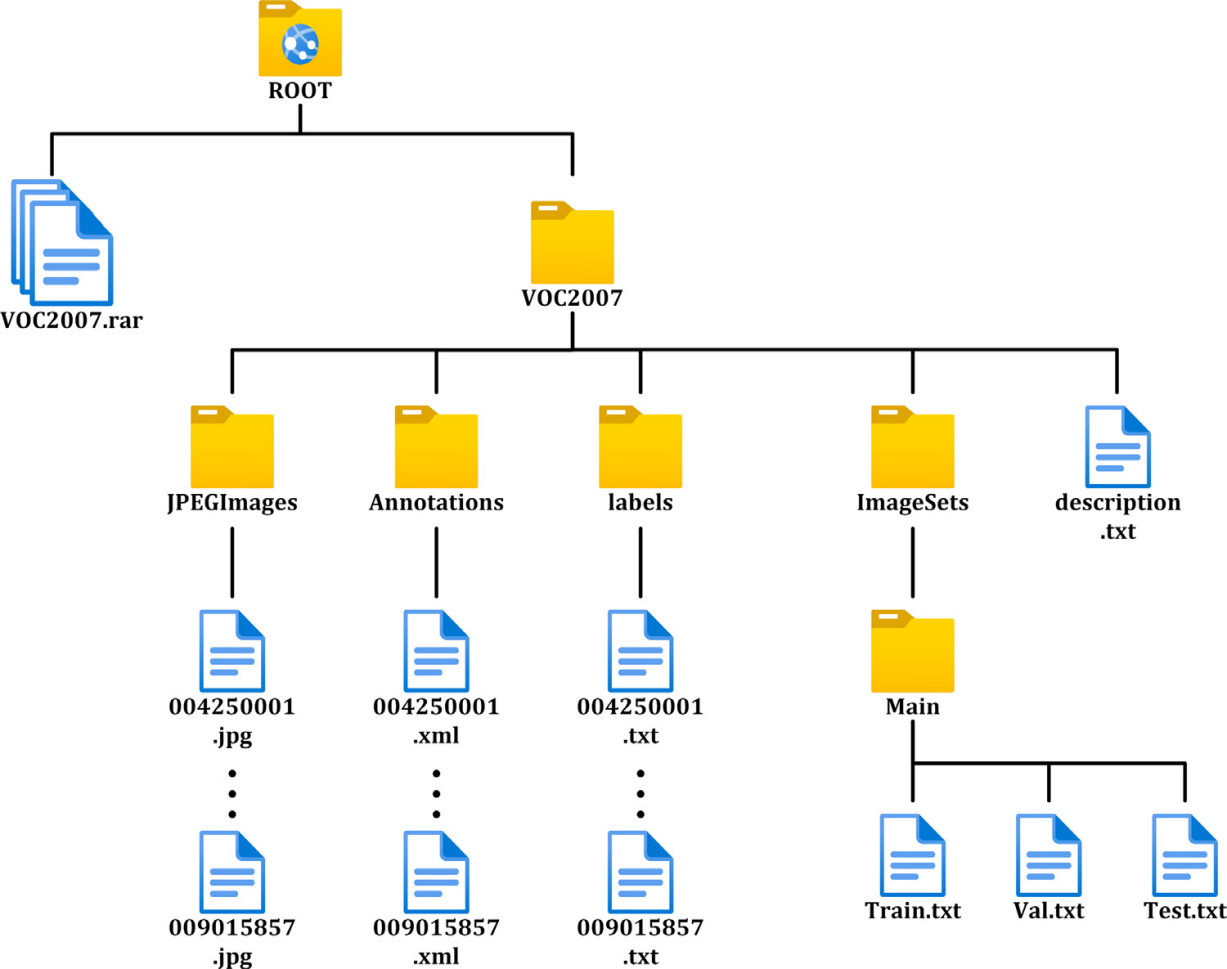
Folder structure.

We constructed the first version of the dataset using the 5857 pomegranate images and then divided them into training and validation sets, as well as a test set. A brief description of the data set in question is shown in Table 4. The folder structure is shown in Fig. 6.

**Table 4:** Brief description of the data set.

No.	Particulars	Description
1	Fruit	Pomegranate
2	Number of growing periods considered	5
3	Original Image	HEIC; 3024*4032 pixels; 72 dpi
4	Converted image	Jpg; 640*480; 96 dpi
5	Annotation file format	XML,TXT
6	Data set size	Size of each image: 33.8-252 KB JPEGImages folder size: 793 MB Annotations folder size: 5.87 MB Labels folder size: 680 KB ImageSets folder size: 62.9 KB VOC2007 folder size: 800 MB VOC2007.rar folder size: 797 MB

The root directory contains two files, a folder named VOC2007 and a zip file named VOC2007.rar. We use two types of storage to make it easier for users to download. The VOC2007 folder is easy for users to browse on the web and view the dataset, while the VOC2007.rar file is easy for users to download directly. Users simply need to download and unzip the file to get the original dataset. The role of each file is shown in Table 5.

**Table 5:** Brief description of the folder.

No.	Name	File formats in the folder	Description
1	VOC2007.rar	rar (in itself)	Conveniently packaged for download
2	VOC2007	-	-
3	JPEGImages folder	jpg	Image files for easy viewing
4	Annotations folder	XML	Labeled file with information such as category, location & size. The model can be used for training.
5	Labels folder	TXT	Labeled file with information such as category, location & size. The model can be used for training.
6	ImageSets folder	TXT (in Mian folder)	It is used to divide the training set, validation set and test set. Contains file names by which the model can find the corresponding file.

Let's continue to explain the VOC2007 folder. The VOC2007 folder contains four main folders, as well as a txt file explaining the description of this dataset, called description.txt. This txt folder briefly describes the file structure of this dataset as well as a description of the dataset's contents.

#### 2.3.1 JPEGImages Folder

It includes all 5857 pictures in jpg format. The folder size is 793 MB. The naming rule of the pictures is 9 Arabic numbers, the first one is 0, the second to the fifth one is the date of shooting, which is convenient for users to check the growth period time; the last 4 bits are the picture number, from 0 to the end of 5857. For example, 007184341.jpg indicates that the image was taken on July 18th and is number 4341 out of all the images.

#### 2.3.2 Annotations Folder

This folder is the annotation file and includes 5857 xml files. The folder size is 5.87 MB. The naming rules are the same as for the JPEGImages folder, with 9 Arabic digits, and correspond to the images in the JPEGImages folder. Each xml file includes the folder, filename, path, source, size, segmented, and object annotation information. object annotation information includes the class name (bud, flower, early-fruit, mid-growth, and mature) and the coordinate information of the real bounding box.

#### 2.3.3 Labels Folder

This folder includes YOLO annotation files in txt format, totaling 5857 files, which correspond to the original image files one by one. The folder size is 680 KB. Each file contains 5 columns of data, the first column is the category, which consists of numbers 0-4, corresponding to the categories of bud, flower5. Column 2.3 is the coordinates of the center point of the real bounding box, and column 4.5 is the length and width of the real bounding box. And the data in columns 2-5 are normalized to values between 0 and 1. The number of rows of data in this file is the number of real bounding boxes.

#### 2.3.4 ImageSets Folder

The folder includes a folder named Main, which contains three txt files: train.txt, val.txt, and test.txt. train.txt contains data numbers of randomly selected training data set, totaling 4683 rows of data, one number per row. val.txt contains data numbers of randomly selected validation data set, totaling 587 rows of data, one number per row. test.txt contains data numbers of randomly selected test data set, totaling 587 rows of data, one number per row. All numbers correspond to the number of the original image.

In summary, the VOC2007 folder contains the training and validation sets, which include a total of 5270 pomegranate images, while the test folder contains the test set, which includes a total of 587 pomegranate images.

## 3 Experimental Design, Materials and Methods

Pomegranate (Punica granatum L.) is a diploid fruit crop (2n = 16) and a member of the Lythraceae family [1,2]. Pomegranate is considered an important fruit crop in semi-arid tropical regions and has a global cultivation area of about 550,000 hectares with a yield of around 6.5 million tons [3]. It is a nutritious and delicious fruit product oriented towards export-oriented agriculture that ensures high returns for growers. It is also considered a strategic crop for ensuring nutritional and livelihood security in water-scarce regions of the world and can help to mitigate climate change to some extent [4].

By applying some computer vision and deep learning techniques, it is possible to recognize and detect pomegranates based on their images, and to detect the growth stages of pomegranates. Traditionally, farmers and crop experts mostly rely on their experience to cultivate plants, but in recent years, technological innovations have helped humans to intelligently manage various plant cultivations within a relatively short time and cost. Nowadays, machine learning disciplines can detect with astonishing levels of accuracy (provided they have enough data available). Moreover, machine learning algorithms can be applied to a range of fields, including agriculture, using low-cost computer hardware.

The dataset is crucial for the success of machine learning, as a good and sufficient dataset can greatly enhance its performance. Machine learning algorithms extract hidden features from the dataset (this is called learning or training the model), and then detect unknown events based on these learned patterns. Therefore, there is a high correlation between the quality of the dataset and the performance of the machine learning system. The quality of the dataset can be measured from aspects such as its size, intra-class consistency, inter-class difference, imbalance of data distribution between different categories, and noise or missing labels in data and labels. Having these characteristics makes the dataset standardized. The dataset must be a real representative of the real scenario where the learning system will be applied; otherwise, the abilities of these models cannot be utilized. Therefore, different detection tasks require different datasets, which makes the dataset itself valuable. However, in terms of real-world datasets for plant growth cycle detection, actual data is not abundant. In this study, we aim to develop a standard, easy-to-use, and publicly available dataset for the pomegranate growth cycle.

### 3.1 Experimental Process

Our main stages of the entire data set preparation process are as follows:

(1) Preparatory work. Research and learn about pomegranates and machine learning.
(2) Physically capture images. Take pictures of pomegranates from trees at different times. As mentioned earlier, we considered a total of 5 cycles.
(3) Validate the dataset images. This step includes image format conversion, cleaning images from background noise, dividing into training and validation sets, adjusting images to the standard shape used by machine learning researchers with the help of human experts, and classifying and labeling them.

Below, we will provide a detailed explanation of the aforementioned steps.

### 3.2 Pre Work

We have found that compared to other fruit categories such as apples, oranges, and bananas, there are relatively few publicly available pomegranate datasets. Furthermore, among all agricultural fruit datasets, datasets specifically focusing on the growth stages are even scarcer. For instance, Arun Kumar R et al. [5] proposed a dataset for pomegranate quality, which involved classifying mature pomegranates after harvesting. Dhanashree K. Barbole et al. [6] introduced a dataset for grape clusters, but it also involved capturing mature grape clusters after harvesting. Angelica Giancaspro et al. [7] assessed the genetic diversity of wild and cultivated pomegranates using microsatellite markers, establishing phylogenetic relationships (genetic similarity and distance) among pomegranate germplasms and comparing genetic clustering with morphological classification. Dineshkumar K et al. [8] presented the Long-read genome sequence data of pomegranate bacterial blight pathogen Xanthomonas citri pv. punicae 119, and so on. As a result, publicly available datasets focusing on the growth stages of pomegranates are extremely limited.

In summary, most agricultural datasets involve destructive sampling of mature fruits or extraction of molecular and genetic components after harvesting. The main contributions of our dataset are as follows: firstly, our images were captured during the growth process of pomegranates, without disrupting their growth. Secondly, we address the scarcity of pomegranate-specific datasets. Lastly, we fill the gap in datasets focusing on fruit growth stages.

Currently, there is no clear classification standard for the growth stages of pomegranates. This study visited professional researchers and fruit growers, and based on some visual characteristics of pomegranates, combined with the different management needs of pomegranates at each stage and relevant research papers [9], classified pomegranates into five different categories: bud, flower, early-fruit, mid-growth, and ripe.

Similarly, we need to know what form of the dataset can be better used by machine learning models to better create the dataset. Therefore, we studied a large amount of related knowledge in the early stage.

### 3.3 Physical Capture of Images

**Fig. 7:**
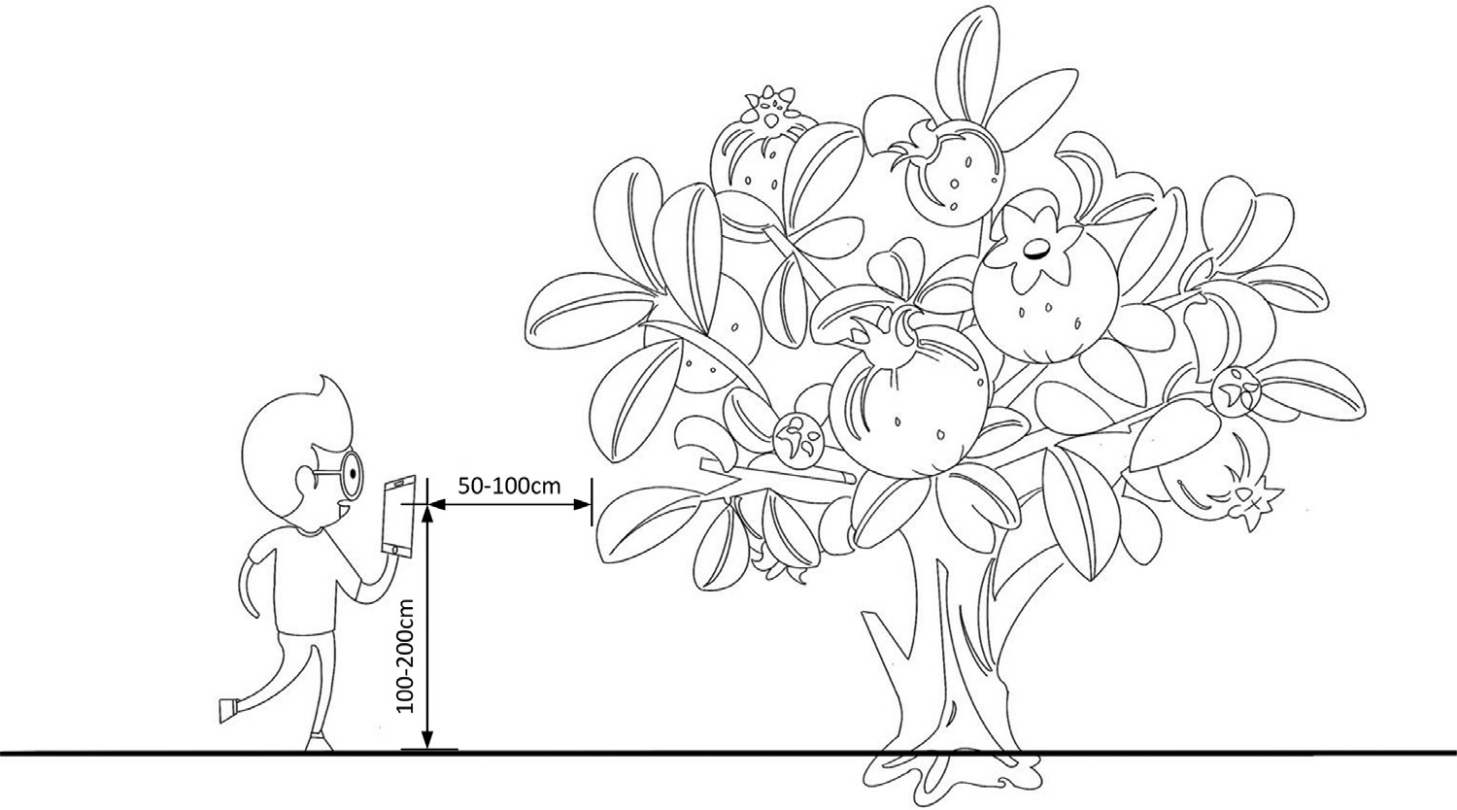
Photo-taking method.

The experiment was conducted in a pomegranate orchard located on the campus of Henan University of Science and Technology in Xinxiang City, Henan Province, China. The experiment was carried out from May 2022 to September 2022, and all pomegranate fruits were allowed to grow in their natural state. The dataset was collected using an iPhone XR camera with a 12 megapixel sensor and f/1.8 aperture lens (1.4 µm pixel size). The rear camera features a CMOS sensor, optical image stabilization, and autofocus. The photos were taken with the normal 1x zoom setting, hand-held by a person at a height of 100 cm-200 cm and a distance of 50 cm-100 cm from the fruit. A brief description of the picture taking is shown in Table 6. The shooting method is shown in Fig. 7.

**Table 6:** Brief description of the camera device.

No.	Particulars	Description
1	Camera manufacturer	Apple
2	Camera model	iPhone XR
3	Lens Model	iPhone XR back camera 4.25mm f/1.8
4	Aperture value	f/1.8
5	exposure time	1/121 s
6	Camera flash	None
7	Monitoring range	4mm
8	Imaging	Size: 3024*4032 pixels
		Resolution: 72 dpi
		Bit depth: 32

### 3.4 Validation of Images in the Dataset

Firstly, the software iMazing Converter developed by DigiDNA Sàrl was used to convert 5875 pomegranate images from HEIC to jpg format while maintaining the original image quality. As the size of the images must be the same for the machine learning model, each captured image was adjusted to a size of 640×480 pixels for better visualization.

After removing some unusable noisy images due to blurriness, scratches, etc., our dataset consists of a total of 5857 pomegranate images.

Next, they divided the dataset into a training set of 4685 images and a validation set of 585 images, as well as a test set of 587 images.

Finally, we used the open-source annotation software LabelImg to annotate the pomegranate dataset, manually annotating all the pomegranates in the collected images as rectangles and labeling them as ``bud'', ``flower'', ``early-fruit'', ``mid-growth'', and ``ripe'', and saved the annotation files in "XML" format. Although we have carefully studied the characteristics of the pomegranate growth cycle ourselves, sometimes we still verify our judgment of image labels with agricultural experts. This makes the quality of the labels more reliable.

### 3.5 Summary and Outlook

The pomegranate growth stage dataset presented here fills a significant gap in the available resources for researchers and agricultural practitioners. The dataset provides high-quality images of pomegranates at various stages of growth, which can be used to study the growth process and develop more effective management practices. The dataset also has the potential to be used to develop machine learning and computer vision algorithms for applications such as automated harvesting and quality assessment. We recognize that the current dataset is limited in its environmental scope, as it was primarily collected in a single orchard in China. In future research, we plan to expand the dataset to include images of pomegranates grown in a wider range of environments, including different climates and weather conditions. This will increase the diversity and applicability of the dataset, making it a more valuable resource for researchers and agricultural practitioners.

We believe that the expanded dataset will have a significant impact on the agricultural sector. By providing a more comprehensive and diverse resource for research, the dataset will help to drive advancements in agricultural technology and improve agricultural production.

## Limitations

None.

## Ethics Statement

The proposed data does not involve any human subjects, animal experiments, or data collected from social media platforms.

## CRediT authorship contribution statement

**Jifei Zhao:** Conceptualization, Methodology, Software, Writing – original draft, Investigation. **Rolla Almodfer:** Supervision, Validation, Writing – review & editing. **Xiaoying Wu:** Validation, Writing – review & editing. **Xinfa Wang:** Validation, Writing – review & editing.

## Data Availability

Pomegranate Images Dataset (Original data) (Mendeley Data).
